# Identification of Olfactory Receptors Responding to Androstenone and the Key Structure Determinant in Domestic Pig

**DOI:** 10.3390/cimb47010013

**Published:** 2024-12-30

**Authors:** Peidong Yang, Tingting Luo, Shuqi Yang, Anjing Zhang, Yuan Tang, Li Chen, Jinyong Wang, Yongju Zhao, Zhining Zhong, Xuemin Li, Ziyin Han, Yupei Zhang, Yue Tang, Jideng Ma, Long Jin, Keren Long, Mingzhou Li, Lu Lu

**Affiliations:** 1 State Key Laboratory of Swine and Poultry Breeding Industry, College of Animal Science and Technology, Sichuan Agricultural University, Chengdu 610000, China; yangpeidong@stu.sicau.edu.cn (P.Y.); luotingting@stu.sicau.edu.cn (T.L.); 2022302137@stu.sicau.edu.cn (S.Y.); tangyuan2@stu.sicau.edu.cn (Y.T.); wakira1230@gmail.com (Z.Z.); lixuemin@stu.sicau.edu.cn (X.L.); ziyinhan@stu.sicau.edu.cn (Z.H.); zhangyupei@stu.sicau.edu.cn (Y.Z.); 202300322@stu.sicau.edu.cn (Y.T.); jideng.ma@sicau.edu.cn (J.M.); longjin@sicau.edu.cn (L.J.); keren.long@sicau.edu.cn (K.L.); 2 Department of Pig Production, Chongqing Academy of Animal Science, Chongqing 402460, China; zhanganjing@stu.sicau.edu.cn (A.Z.); lichen5696@163.com (L.C.); kingyou@vip.sina.com (J.W.); 3 College of Animal Science and Technology, Southwest University, Chongqing 402460, China; zyongju@163.com

**Keywords:** pig, olfactory receptors, androstenone, transcriptome, OR7D4

## Abstract

Olfactory receptors (ORs) are members of the transmembrane G protein-coupled receptor superfamily, playing a crucial role in odor recognition, which further mediates crucial biological processes in mammals. In sows, androstenone can trigger sexual behaviors through olfaction, but the underlying mechanism remains to be explored. To efficiently and accurately screen pig olfactory receptors responding to androstenone and the key structure determinant, we adapted the high-throughput RNA-seq strategy to screen the altered genes upon androstenone treatment in the olfactory epithelium of pigs, yielding 1397 downregulated genes. Of which, 15 OR genes and 49 OR-like genes were candidate androstenone-responsive genes, and 5 ORs (OR2D2, OR8D1, OR8D2, OR10Z1 and OR7D4) were proven as responsible for androstenone-mediated olfaction in vitro. Among the five ORs, pig OR7D4 has the highest level of androstenone response. To further find the structural determinant, we performed ligand-binding cavity analysis on pig OR7D4 with androstenone, predicted seven potential structural sites and further confirmed that F178 and T203 are the key sites for recognizing androstenone. Nevertheless, the natural non-synonymous mutation M133V (rs696400829) of pig OR7D4 was proven to significantly impair the respondence to androstenone. This is the first time the ORs responding to androstenone in pigs and the key structural determinant of pig OR7D4 were identified, which highlights the significance of investigating the role of OR7D4 in pig reproduction performance in the future.

## 1. Introduction

Olfaction mediates vast behaviors that are crucial for animals’ survival, such as hunting, sexual behavior (sexual exploration, mating, etc.) and maternal behavior (identification of offspring) [1,2]. Mammalian olfaction greatly relies on a large pool of olfactory receptors (ORs), mediating the conversion of chemical signals into nerve impulses [3,4]. ORs are seven-helix transmembrane proteins located in the olfactory epithelium (OE) as members of the G protein-coupled receptors (GPCRs) superfamily [5]. Upon odor molecules specifically binding to the corresponding ORs, the resulting electronic signals are transmitted via axons to the olfactory bulb (OB) for further integration and then transmitted to the olfactory cortex (OC) and other higher brain structures, resulting in the sense of smell [4].

One olfactory receptor neuron (ORN) expresses only one OR gene in a pair of homologous chromosomes, and each odor can be recognized by varieties of ORs [6,7,8,9,10]. Mammals carry a large number of OR genes, forming diverse receptor combinatorial codes to ensure their odor perception ability. OR de-orphanization is the first step to research OR-mediated olfactory information transduction [11]. Early de-orphanization efforts on ORs were mainly achieved by detecting the response of isolated ORNs to specific odors [12]. However, such solution is characterized by low throughput, which is not applicable to large-scale OR de-orphanization. To address this problem, some studies have been devoted to the establishment of OR heterologous cell expression to achieve large-scale OR de-orphanization. There is a key constraint for constructing heterologous cells that stably express OR: after the integration of OR gene-containing plasmid in heterologous cells, the OR protein will be generated and folded in the endoplasmic reticulum and further modified in the Golgi apparatus before reaching the cell membrane for functional expression. However, the heterogeneous expression of OR tends to be retained in the endoplasmic reticulum during transporting and ultimately degraded due to reasons such as the insufficient folding ability of OR in heterologous cells [13]. Zhuang et al. [14] (2007) circumvented this problem in an ingenious way and established a HEK293-derived cell line, Hana3A, for the stable heterologous expression of OR.

Heterologous expression of OR accelerates the de-orphanization of this protein family. However, ORs responding to specific odor in vitro may not respond to the same agonist in vivo [15]. To address this issue, several studies have attempted to validate the responses of ORs to ligands using approaches that combine in vivo assays and heterologous expression. For example, Jiang et al. [16] (2015) isolated ORNs activated by odor after stimulation in vivo, invoked the transcriptome data to screen for ORs potentially responsive to a specific agonist and validated this in vitro. Von et al. [17] (2015) found that the expression of ORs responding to a specific agonist was significantly downregulated after stimulation with an agonist.

As an important domestic animal, only a few studies have been focused on the field of olfaction in domestic pigs (*Sus scrofa*). Pigs have a well-developed sense of smell, which is partially owed to their great number of ORs. Of which, only 11% are pseudogenes, the lowest proportion of any known species [5]. Androstenone, known as the first pheromone found in pigs, was first identified in boars’ saliva [18]. As a steroidal substance secreted by the testicular tissue of post-pubertal boars, androstenone is usually released into the blood and accumulates in the salivary glands of boars [19]. The salivary glands of boars contain large amounts of androstenone, which is released in large quantities into the saliva after dissociation from specific binding proteins. When a boar is in estrus, significantly aggressive and sexually impulsive, it chews its teeth vigorously, resulting in the formation of large quantities of frothy saliva in the oral cavity [20]. Androstenone in saliva is uniquely attractive to sows in estrus. Upon sensing androstenone, sows will actively arch their backs in a ready-to-mate position [21]. This property of androstenone makes it the main active ingredient in the artificial insemination product “Boarmate”, which is widely used to check the state of sexual receptivity of sows in estrus in order to confirm the right time for artificial insemination. More recently, androstenone has also been used in the estrous induction of sows [22]. However, the mechanism by how androstenone is recognized in pigs is not clear. To efficiently and accurately screen pig olfactory receptors responding to androstenone, we hypothesized that the expression of androstenone ORs in pig OEs would undergo significant downregulation after androstenone stimulation. Here, we combined RNA sequencing (RNA-seq) with a heterogenous expression system to identify the ORs responding to androstenone in pigs. Nevertheless, to find the key structure determinant, the key amino acids and natural single-nucleotide polymorphisms (SNPs) affecting the responding activity of the ORs to androstenone were also verified. The results of this study establish a theoretical basis for subsequent studies aiming to correlate ORs with pigs’ reproductive performance.

## 2. Materials and Methods

### 2.1. Animal Experiment

All animals were treated humanely during husbandry and sampling, and the animal care and experiment were approved with permission (Permission number: 20230089) by the Experimental Animal Ethics Committee of Sichuan Agricultural University. The sows were the offspring of Landrace (♂) and Rongchang pigs (♀), aged 8 months, kept at the Academy of Animal Husbandry, Chongqing, China. All sows were in estrus and in good health. Four sows were randomly divided into the androstenone treatment group and control group, with two in each group. In the androstenone treatment group, the Boarmate (KERBL, Buchbach, Germany, 4505675) gifted from the Chongqing Academy of Animal Science was sprayed on the nasal cavity before slaughter, while the control group received no solution treatment before slaughter. After 30 min, the sows were sacrificed, and OEs from the left and right side were collected from each sow. The samples were snap-frozen in liquid nitrogen before being stored at −80 °C. All animals were treated humanely during husbandry and sampling.

### 2.2. RNA-Seq

RNA was extracted using Trizol (Invitrogen, Carlsbad, CA, USA, 15596018), according to the manufacturer’s instructions, and tested for concentration and purity using a NanoDrop 2000 (Thermo Fisher, Waltman, MA, USA) and bioanalyzer (Agilent Technologies, Palo Alto, CA, USA). RNA that passed the assay (1.8 ≤ OD260/OD280 ≤ 2.0, RIN ≥ 8) were used for high-throughput sequencing library construction.

RNA-seq libraries were prepared following the NEBNext^®^ Ultra^TM^ RNA Library Prep Kit (New England Biolands, Lpswich, MA, USA, 7530). The paired-end RNA-seq libraries were further sequenced by the DNBSEQ-T7 platform, sequencing data volume 10 Gb, producing an average of 246 million 150 bp paired-end raw reads (Novogene, Tianjin, China). We applied fastp v.0.23.1 for base quality filtering of raw data to obtain clean data [23]. We downloaded pig genome (Sus_scrofa.Sscrofa11.1.cdna.all.fa) and annotation files (Sus_scrofa.Sscrofa11.1.108.gtf) via Ensembl (https://asia.ensembl.org, accessed on 17 February 2023) and performed read alignment and quantification on clean data using Kallisto v.0.48.0 [24]. The differentially expressed genes (DEGs) were identified by edgeR [25], setting *p* < 0.05 and |log2fold change| ≥ 1 as the cutoff. We performed the Gene Ontology biological process and KEGG pathway enrichment analysis by R package clusterProfiler v.4.12.2. The RNA-seq data were submitted to the Gene Expression Omnibus (GEO) database: GSE279037.

### 2.3. Cloning of Olfactory Receptors

We utilized the HiScript III 1st Strand cDNA Synthesis Kit (Vazyme, Nanjing, China, R312-01) to generate cDNA, referring to its instructions. The primers used for fragment amplification were designed using Primer 5 (Table S1), and the CDS regions corresponding to the ORs were amplified using 2 × Phanta^®^ Max Master Mix (Vazyme, Nanjing, China, R312-01). RHO- pCI, pCRE-Luc, pSV40-RL, RTP1S and M3 were generously gifted from Prof. Zhuang Hanyi (Shanghai Jiao Tong University School of Medicine). We inserted the CDS regions into the RHO-pCI plasmid. Briefly, we used restriction endonucleases NotI (New England Biolands, Lpswich, MA, USA, R0189V) and MluI (New England Biolands, Lpswich, MA, USA, R0198V) to cleave the RHO-pCI plasmid and the CDS regions of the ORs, respectively. We used T4 DNA Ligase (New England Biolands, Lpswich, MA, USA, M0202V) for ligation. The ligated product was transformed into *E. coli* (Tsingke Biotech, Beijing, China, TSC-C01). After 24 h of incubation at 37 °C, a monoclone was selected for culture and sent to be sequenced (Tsingke Biotech, Beijing, China). In order to obtain the mutation plasmid, we used the Mut Express MultiS Fast Mutagenesis Kit V2 (Vazyme, Nanjing, China, C215) to perform point mutation in the CDS region of the ORs. The primers are shown in Table S2.

### 2.4. Cell Culture

Hana3A cells were gifted from Prof. Zhuang Hanyi (Shanghai Jiao Tong University School of Medicine) and were cultured in minimal essential medium (Gibco, Waltham, MA, USA, 11095080) containing 10% fetal bovine serum (Gibco, Waltham, MA, USA, 10091155) with the addition of a final concentration of 100 μg/mL of penicillin–streptomycin (Beyotime, Shanghai, China, C0222) and 1 μg/mL of puromycin (Solarbio, Beijing, China, P8230). Cells were inoculated at a density of 1 × 10^5^/mL in T25 culture flasks (NEST, Wuxi, China, 707003) and cultured at 37 °C, 5% CO_2_ and passaged every 2 days.

### 2.5. Transfection

Hana3A were plated in T25 culture flasks; after 2–3 passages, cells were seeded in 96-well plates at a density of 2 × 10^6^/mL. Plasmid transfection was performed after 24 h of incubation at 37 °C, 5% CO_2_ when the confluence was 50–70%. For 96-well plates, 0.5 μg of RHO-OR, 0.1 μg of pCRE-Luc, 0.1 μg of pSV40-RL, 0.1 μg of RTP1S and 0.05 μg of M3 were transfected per well [26]. Transfections were performed according to the instructions of Lipofectamine 3000 (Thermo Fisher, Waltman, MA, USA, L3000150). Briefly, the transfected plasmids were premixed with P3000 reagent and added to minimal essential medium for transfection. Subsequently, Lipofectamine 3000 was premixed with serum-reduced medium (Gibco, Waltman, MA, USA, 31985070). The mixture obtained from the two steps above was mixed, blown gently and incubated at room temperature for 10 min to obtain the transfection mixture. Then, 10 μL/well of the transfection mixture was added drop by drop per well, and the plate was gently panned to mix the mixture before being incubated at 37 °C with 5% CO_2_ for 24 h.

### 2.6. Stimulation and Measuring Activity of Olfactory Receptors

After 24 h of transfection, the 96-well plate was removed from the incubator, the medium was aspirated, and 50 μL of serum-reduced medium was added to each well and incubated for 30 min at 37 °C, 5% CO_2_ [27]. The androstenone reserve of 10 mM was sequentially diluted with serum-reduced medium into androstenone stimulation solution of 100 μM and 200 μM. Another set of serum-reduced medium without androstenone was used for the negative control group. The medium in the cell culture plate was aspirated, and 25 μL of stimulation solution was added to each well and incubated at 37 °C, 5% CO_2_ for 4 h. We evaluated the responsiveness of the ORs according to the instructions of the Dual-Glo^®^ Luciferase Assay System (Promega, Madison, WI, USA, E2920). The 96-well plate was removed from the incubator and equilibrated to room temperature. Then, 75 μL of firefly luciferase luminescence assay solution was added to each well, and the firefly luciferase luminescence values were measured on a Promega Luminescence Detector (Promega, Madison, WI, USA) after 10 min of incubation at room temperature. After, 75 μL of renilla luciferase luminescence assay solution was added to each well, and the renilla luciferase luminescence value was measured after incubation at room temperature for 10 min. The response of each OR to androstenone was normalized by calculating the ratio of the firefly luciferase luminescence value to the renilla luciferase luminescence value.

### 2.7. Protein Sequence Analysis

We used AlphaFold2 (https://alphafoldserver.com) to simulate the 3D structure of the ORs [28,29]. The androstenone molecular structure was obtained through PubChem (https://pubchem.ncbi.nlm.nih.gov). Then, we used ProteinPlus to predict the ligand-binding cavity of pig OR7D4 [30,31,32]. AutoDock Tools v.1.5.6 was used to generate pdbqt files, and molecular docking was implemented using AutoDock v.4.2.6. We made the molecular docking box cover the entire OR protein to ensure that all sites were involved in the simulated molecular docking. The number of dockings was set to 100. The results were visualized using Pymol v.2.5.0 [33,34].

The ligand-binding cavity of pig OR7D4 predicted was analyzed by calculating the Grantham distance [35] to screen relatively conserved residues. We used Clustalx v.2.1 to align the amino acid sequences of all annotated pig ORs, and the pairwise Grantham distances of each site in the ligand-binding cavity of pig OR7D4 across these ORs were calculated, and their mean was computed. The mean value of pairwise Grantham distances of ligand-binding cavity of pig OR7D4 in the five androstenone ORs was also calculated. If the mean value of the pairwise Grantham distance of a residue within the 5 androstenone ORs was significantly lower than the value within all the annotated pig ORs (*p* < 0.05), the residue was considered to be more conserved among the androstenone ORs.

### 2.8. Statistical Analysis

Data were shown as mean ± SEM using SPSS v.19.0 for statistics of variance. We calculated the different significance by *t*-test or Mann–Whitney test, *p* < 0.05 was considered a significant difference and *p* < 0.01 was considered highly significant.

## 3. Results

### 3.1. Transcriptomic Changes in Pigs’ Olfactory Epithelium Treated with Androstenone

**Figure 1:**
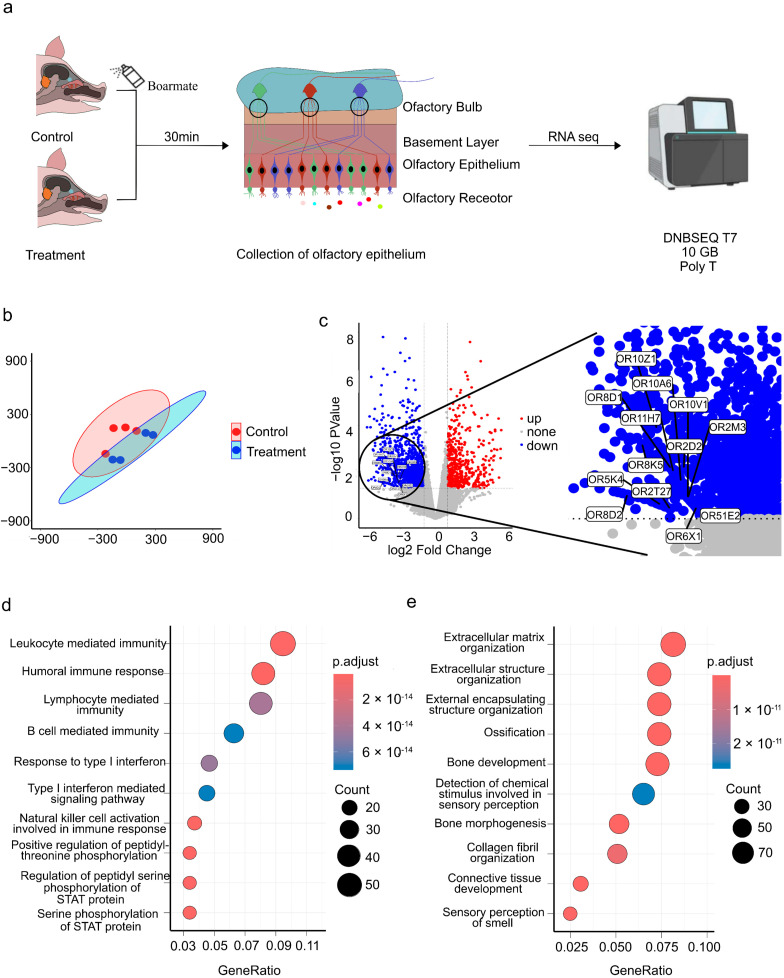
Transcriptomic change in pigs’ olfactory epithelium upon androstenone treatment. (**a**) Flowchart of sampling and transcriptome sequencing. (**b**) tSNE clustering of RNA-seq samples; red and blue represent samples from the control group and the androstenone treatment group, respectively. (**c**) Volcano plot of DEGs. (**d**) Top 10 GO terms of the up-DEGs. (**e**) Top 10 GO terms of the down-DEGs.

To investigate the effect of androstenone on pigs’ OE, we sprayed Boarmate on sows’ nasal cavities, which mainly contains androstenone. After 30 min, the OE were collected and further applied for RNA-seq, yielding 294.78 Gb raw data from eight transcriptomic samples (Figure 1a, Table S3). The eight samples were well clustered into two groups, the control group and the androstenone treatment group (Figure 1b), indicating androstenone altered the global gene expression of pigs’ OE. Setting |log2(FC)| ≥ 1 and *p* < 0.05 as the cutoff, we identified 1993 DEGs. Of which, 596 and 1397 DEGs were upregulated or downregulated in the androstenone treatment group compared to the control group, respectively (Figure 1c). The downregulated DEGs were significantly enriched in signaling pathways or biological processes related to olfactory perception, such as olfactory transduction (hsa04740), detection of chemical stimulus involved in sensory perception (GO: 0050907) and sensory perception of smell (GO: 0007608) (Figure 1d,e and Figure S1, Tables S4–S7), indicating that androstenone can cause significant expression change of genes related to olfactory perception in pigs’ OE.

### 3.2. Validation of Olfactory Receptors Responding to Androstenone

Von et al. [17] (2015) demonstrated that olfaction activation causes the transcriptomic inhibitory effect on the OR genes that are in charge of responding to the odor. Thus, we screened the candidate OR genes responding to androstenone from the downregulated DEGs. We acquired 15 OR genes (Table S8) and 49 OR-like genes (Table S9) that are downregulated in the androstenone treatment group compared to the control group. It is worth mentioning that Zhuang et al. (2007) [14] proposed that OR7D4 with eight other ORs was activated by androstenone in humans. We confirmed that the pig homologous gene (ENSSSCG00000058680, hereafter named pig *OR7D4*) had significantly lower expression levels in the samples of the androstenone treatment group compared to the control group.

**Figure 2:**
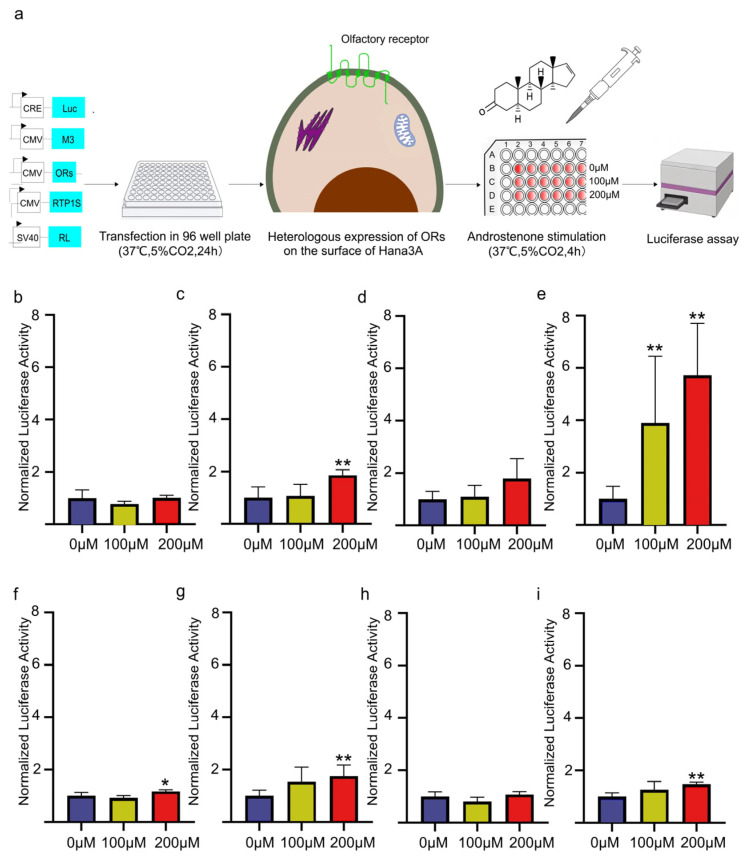
Response of the olfactory receptors to different concentrations of androstenone. (**a**) Flowchart of the heterologous expression and functional assay of ORs. (**b**) Response of the control group (not transfected with ORs) to androstenone. (**c**) Response of OR2D2 to androstenone. (**d**) Response of OR6X1 to androstenone. (**e**) Response of OR7D4 to androstenone. (**f**) Response of OR8D1 to androstenone. (**g**) Response of OR8D2 to androstenone. (**h**) Response of OR10V1 to androstenone. (**i**) Response of OR10Z1 to androstenone. The different colours of columns represent the different concentrations of adrostenone. The error bar represents SEM, * represents *p* < 0.05 and ** represents *p* < 0.01.

Using the heterogenous expression system, we successfully cloned OR2D2, OR6X1, OR8D1, OR8D2, OR10V1, OR10Z1 and pig OR7D4 and expressed these ORs in Hana 3A cells (Figure 2a). Stimulated with androstenone in different concentrations, the results indicated that OR2D2, OR7D4, OR8D1, OR8D2 and OR10V1 can be activated by androstenone, while OR6X1 and OR10V1 were unable to be activated in the 200 μM androstenone treatment (Figure 2b–i). Of which, OR7D4 showed a significant response when stimulated with 100 μM androstenone.

### 3.3. Screening Key Sites of the Pig OR7D4 Response to Androstenone Based on Amino Acid Conservation

**Figure 3:**
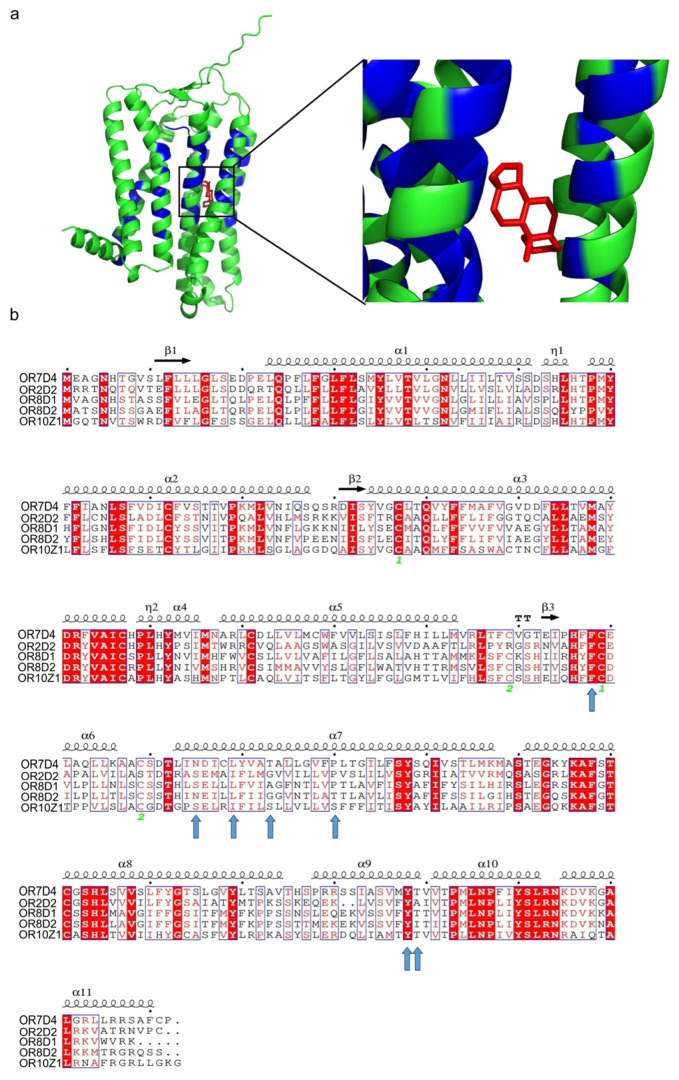
Potential key sites mediating the response to androstenone of pig OR7D4. (**a**) Molecular docking plot. Simulated docking results of pig OR7D4 with androstenone molecules marked in red. Binding cavity predicted are marked in blue. (**b**) Alignment of androstenone ORs of pigs. Relatively conserved sites in the binding cavity of pig OR7D4 are marked by arrows. Highly conserved loci are marked in red, with 10 loci between each black dot. Secondary structures are shown above the sequence.

Given pig OR7D4 shows the most significant response to androstenone, we further aimed to find the structural determinant. The 3D structure of pig OR7D4 was simulated, and the ligand-binding cavity was predicted by ProteinPlus [30,31,32]. We found that most of the residues in the ligand-binding cavity are located on transmembrane (TM) helix 2-7 and extracellular loop (ECL) 2 (Table S10). Moreover, the molecular docking results also indicated the interaction region located in the cavity formed by the TMs and ECL2 (Figure 3a).

**Figure 4:**
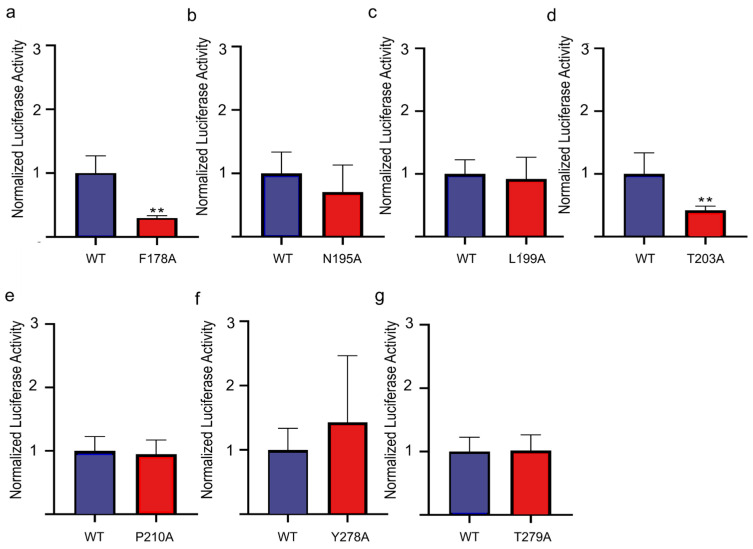
Evaluation of potential key sites mediating the response to androstenone of pig OR7D4. (**a**) Response of pig OR7D4-F178A compared to that of pig OR7D4 wild type. (**b**) Response of pig OR7D4-N195A compared to that of pig OR7D4 wild type. (**c**) Response of pig OR7D4-L199A compared to that of pig OR7D4 wild type. (**d**) Response of pig OR7D4-T203A compared to that of pig OR7D4 wild type. (**e**) Response of pig OR7D4-P210A compared to that of pig OR7D4 wild type. (**f**) Response of pig OR7D4-Y278A compared to that of pig OR7D4 wild type. (**g**) Response of pig OR7D4-T279A compared to that of pig OR7D4 wild type. The column height represents the response level of androstenone in the wild type control group (blue) and different mutation groups (red) when simulated with 200 μM androstenone, and the values of each group are presented as the ratio of the values detected relative to the control group. The error bar represents SEM. ** represents *p* < 0.01.

Next, we hypothesized that the key residues mediating the response of ORs to androstenone are relatively conserved among the series of androstenone-activated ORs due to being activated by the same odor [16]. Therefore, a total of seven potential androstenone relatively conserved sites were screened within the ligand-binding cavity of pig OR7D4 with the potential to mediate the response to androstenone (Figure 3b, Figures S2 and S3). These sites with relatively high similarity are all located in the transmembrane helixes of pig OR7D4, as well as in the second extracellular loop, with four located on TM5 and two located on TM7 and one located on ECL2 (Figure S3). Last, alanine scanning of these seven loci further confirmed that F178A and T203A significantly decreased the response of pig OR7D4 to androstenone in vitro, which suggests they are the key structural determinants of pig OR7D4 (Figure 4).

### 3.4. Single-Nucleotide Polymorphisms Affect OR7D4 Responding to Androstenone

**Figure 5:**
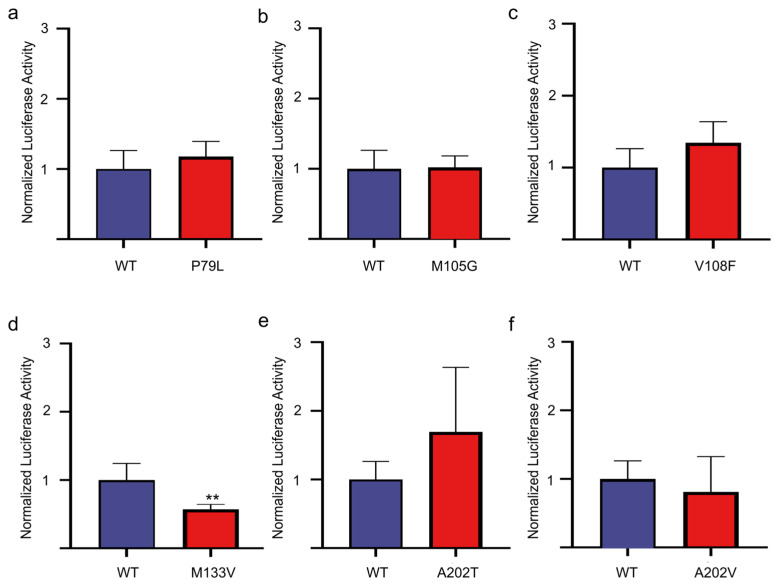
The effect of polymorphism sites caused by different non-synonymous SNPs on the response level of pig OR7D4 to androstenone. (**a**) Response of pig OR7D4-P79L compared to that of pig OR7D4 wild type. (**b**) Response of pig OR7D4-M105G compared to that of pig OR7D4 wild type. (**c**) Response of pig OR7D4-V108F compared to that of pig OR7D4 wild type. (**d**) Response of pig OR7D4-M133V compared to that of pig OR7D4 wild type. (**e**) Response of pig OR7D4-A202T compared to that of pig OR7D4 wild type. (**f**) Response of pig OR7D4-A202V compared to that of pig OR7D4 wild type. The column height represents the response level of androstenone in the wild type control group (blue) and different mutation groups (red) when simulated with 200 μM androstenone, and the values of each group are presented as the ratio of the values detected relative to the control group. The error bar represents SEM. ** represents *p* < 0.01.

Previous studies have shown that non-synonymous SNPs within the OR genes may have a significant impact on olfactory perception [36,37]. We thus searched the variation database and found a total of 42 non-synonymous SNPs in the CDS region of pig *OR7D4* (Table S11). Given P79 and A202 are part of the ligand-binding cavity of human OR7D4, which affects the response of androstenone [38], M105 and G108 are members of the 22 amino acid residues that make up the ligand-binding cavity of all ORs, as proposed by Man et al. [39] (2004), and M133 was proven to strongly affect the androstenone perception in humans [36], so we further evaluated the effect of these mutation on responding to androstenone. As presented in Figure 5, only M133V significantly reduced the response of OR7D4 to androstenone, while no significant difference was found after the mutation of the rest of the loci.

## 4. Discussion

In the present study, 1993 DEGs were detected in the androstenone-treated OE of sows. Of which, many of the genes downregulated in the androstenone treatment group were related to the adaptation of the olfaction system to external stimulation, which indicates the success of the pig model for androstenone treatment. For instance, *SEMA7A* is thought to play a key role in axon formation, and the significant downregulation of this gene after androstenone treatment may be related to the adaptation of the olfaction system to the odor environment by regulating the axon guidance system [40]. Specifically, the downregulated DEGs can be enriched in biological processes or signaling pathways related to olfaction signal transmission. Consistent with Von et al. [17] (2015) and Koerte et al. [41] (2018), we confirmed that odor activation causes inhibition in the expression of the corresponding ORs. In addition to the ORs, we also noted that the expression of *OBP2A* and *OBP2B*, which are associated with odor binding, were significantly downregulated after androstenone treatment. Mammalian OBP is regarded as a kind of soluble protein specializing in pheromone communication that binds to airborne volatile pheromones and transports them to ORs, thus acting as a carrier of odor from the external environment to the olfaction system [42]. We hypothesized that the downregulation of the OR and OBP genes after androstenone treatment may be the result of the adaptation of the olfaction system to external stimulation, a process that desensitizes the olfaction system to androstenone input over a short period of time, which is analogous to the calcium-mediated inhibition of the cyclic nucleotide-gated (CNG) channel [43,44,45,46]. Horgue et al. [45] (2022) reported that the expression of olfactory conductance cascade-related genes in mice, such as, *Cnga2*, *Cngb1b*, etc., were mostly downregulated after being stimulated with an agonist. However, this phenomenon was not found in this study. This may be due to the difference in the method of odorant expose between our two groups.

We selected seven genes for the androstenone response evaluation in vitro, of which six were Ensembl-annotated OR genes and one was an ortholog of the human androstenone-responsive OR *OR7D4*. A total of five of the seven ORs were activated by androstenone, and two showed an elevated response to androstenone, though not significant. The extent of the response to androstenone of the seven ORs in vitro did not appear to be consistent with the transcriptome data, which may be due to the inconsistency of the cellular experimental conditions with the nasal environment: unlike binding to ORs expressed on the membrane of heterologous cells, the odor tends to form nasal mucus with OBP and undergo complex chemical modifications mediated by a variety of metabolic enzymes therein before interacting with ORs in the nasal cavity, leading to the possibility that the relationship between odor and ORs expressed in heterologous systems may be inconsistent with the realities in vivo [47]. In addition, the distribution of different ORs in different locations in the nasal cavity may allow ORs to be exposed to different concentrations of androstenone during stimulation. *OR10V1*, which was significant downregulated after the stimulation, was not activated by androstenone in vitro. This phenomenon may be due to different reasons: androstenone in the nasal cavity may act on OR10V1 at a concentration that exceeds the maximum stimulatory concentration in Hana3A. In addition, it is possible that androstenone produces metabolites in vivo and activated OR10V1 [48].

Among the androstenone-responsive ORs we detected, pig OR7D4 responded the most to androstenone. When stimulated with 100 μM androstenone, OR7D4 responded significantly, and none of the other ORs were activated, suggesting that OR7D4 may play a key role in the detection of androstenone at low concentrations. OR2D2, OR8D1, OR8D2 and OR10Z1 were activated when the concentration of androstenone was raised to 200 μM, but the responses of those ORs were not as significant as OR7D4. This phenomenon is consistent with the description of the OR-mediated odor recognition reported, whereby odors are recognized by a group of ORs during sense of smell formation, and different concentrations of the same odor activate different ORs, with higher concentrations activating extra ORs [49]. Bolding et al. (2018) reported that, when an odor reaches the ORNs, those ORs that are the most sensitive would be activated first and transmit a signal to the piriform cortex (PCx). The signal from other, less responsive, ORs will be suppressed via neural inhibition in the OB [50,51,52]. This phenomenon implies that the recognition of a particular odor relies on a set of the most sensitive ORs [52,53]. McClintock et al. [49] (2020) reported that those sensitive ORs that play a key role in the recognition of a given odor will be first activated during the recognition of an odor, which increases in response to the odor as the concentration rises. Pig OR7D4 showed the most sensitive response to androstenone, which increased with the increasing androstenone concentration. Thus, we inferred that pig OR7D4 may play a key role in the perception of androstenone.

The cavities with ligand-binding properties on the surface and inside proteins are called ligand-binding cavities, and the amino acid residues surrounding them determine an OR’s physicochemical characteristics [53]. We modeled the 3D structure of pig OR7D4 and generated the ligand-binding cavity, and the results were consistent with our expectation that the residues that make up the ligand-binding cavity of pig OR7D4 are located on TM2-TM7, as well as ECL2 [39,54,55]. We hypothesized that the key residues in the ligand-binding cavities of ORs responsible for recognizing specific odors are more conserved, and during the ligand-binding cavity conservation analysis of pig OR7D4, obtained a total of seven key residues that may be crucial for androstenone recognition. The dual-luciferase report assay showed that F178, which is located on ECL2 of pig OR7D4, which is a close neighbor to the highly conserved Cys site on ECL2, significantly impacted the androstenone responsiveness of pig OR7D4. Wheatley et al. [56] (2012) reported the presence of a ligand recognition “hotspot” region near the highly conserved Cys on ECL2 of class A GPCRs, where amino acid residues may mediate the response of ORs to odors. Furthermore, residues on ECL2 are located in the uppermost part of the ligand-binding cavity that are responsible for regulating the volume and shape of the ligand-binding cavity, maintaining the hydrophobic environment and are important for ligand selection in ORs [57]. In addition, we found that T203, the homologous site of T203 in human OR7D4 that is involved in ligand-binding cavity, showed a significant effect on the response to androstenone of pig OR7D4.

In addition, among our predicted key sites for androstenone responding, Y278 and T279 are located close to each other and in the vicinity of the conserved motif PMLNPFIY on TM7. Ryu et al. [58] (2017) reported that the axon terminals of ORs with this conserved motif mainly project on the dorsal region of the OB, which tends to play a key role in the processing of odor information, while the relationship of PMLNPFIY and odor perception remains to be explored. Our study suggested that Y278 and T279 had no significant effect on the function of pig OR7D4, so the effect of this motif on the function of ORs remains to be elucidated.

Nevertheless, it is worth mentioning that we found that the amino acid substitution M133V (rs696400829) of pig OR7D4 significantly affected its response to androstenone. Zhuang et al. (2007) [14] found that the amino acid substitution T133M on human OR7D4 severely impaired OR function. Both sides do not belong to the predicted ligand-binding cavity of ORs, yet still exert an effect on the response of ORs to androstenone. Such phenomenon has also been reported in studies analyzing OR loci. Some loci, although far from the ligand-binding cavity, may also be involved in the metamorphosis process of ORs, which, in turn, affects OR-mediated olfactory signaling [38].

It is worth mentioning that all samples in our study were collected from pigs receiving androstenone stimulation 30 min previous, and the transcriptome changes of pig OE at different time points after androstenone treatment need to be further investigated. Moreover, androstenone ORs other than OR7D4 are far less responsive to androstenone; the reason why OR7D4 showed higher sensitivity to androstenone needs to be elucidated in the future, which will further clarify the mechanism of ORs’ recognition of androstenone.

## 5. Conclusions

Here, we combined RNA-seq with a heterogenous expression system to identify the ORs responding to androstenone in pigs. Five ORs (*OR7D4*, *OR2D2*, *OR8D1*, *OR8D2* and *OR10Z1*) were proven responsive to androstenone-mediated olfaction. Additionally, the key amino acids (F178 and T203) and natural SNPs (rs696400829) affecting the responding activity to androstenone of OR7D4 were also verified. The results of this study establish a theoretical basis for subsequent studies aiming to correlate ORs with pigs’ reproductive performance.

## Data Availability

The RNA-seq data were submitted to the Gene Expression Omnibus (GEO) database: GSE279037.

## Supplementary Materials

The following supporting information can be downloaded at https://www.mdpi.com/article/10.3390/cimb47010013/s1: Table S1: Primers designed for CDS of ORs; Table S2: Mutant primer sequences; Table S3: Summary of sequencing data; Table S4: GO Ontology annotation of upregulated genes in the androstenone treatment group compared to control group; Table S5: GO Ontology annotation of downregulated genes in the androstenone treatment group compared to control group; Table S6: KEGG pathway enrichment analysis of upregulated genes in the androstenone treatment group compared to control group; Table S7: KEGG: pathway enrichment analysis of downregulated genes in the androstenone treatment group compared to control group; Table S8: Downregulated OR genes in the androstenone treatment group compare to control group; Table S9: Downregulated OR-like genes in the androstenone treatment group compared to control group; Table S10: Ligand-binding cavity residues of pig OR7D4; Table S11: Missient variant in pig OR7D4; Figure S1: Top 10 KEGG terms of the DEGs; Figure S2: Seven amino acid sites relatively conserved in olfactory receptors responsive to androstenone in binding cavity of pig OR7D4; Figure S3: Potential key sites mediating pig OR7D4 response to androstenone.
