# Supplementary material for: Identification of Olfactory Receptors Responding to Androstenone and the Key Structure Determinant in Domestic Pig
Source: Curr Issues Mol Biol. 2024 Dec 30;47(1):13. doi: 10.3390/cimb47010013 (PMC11763519; doi:10.3390/cimb47010013)
Supplement: Supplementary file 1 [file cimb-47-00013-s001.zip › Figure S1.pdf]

a

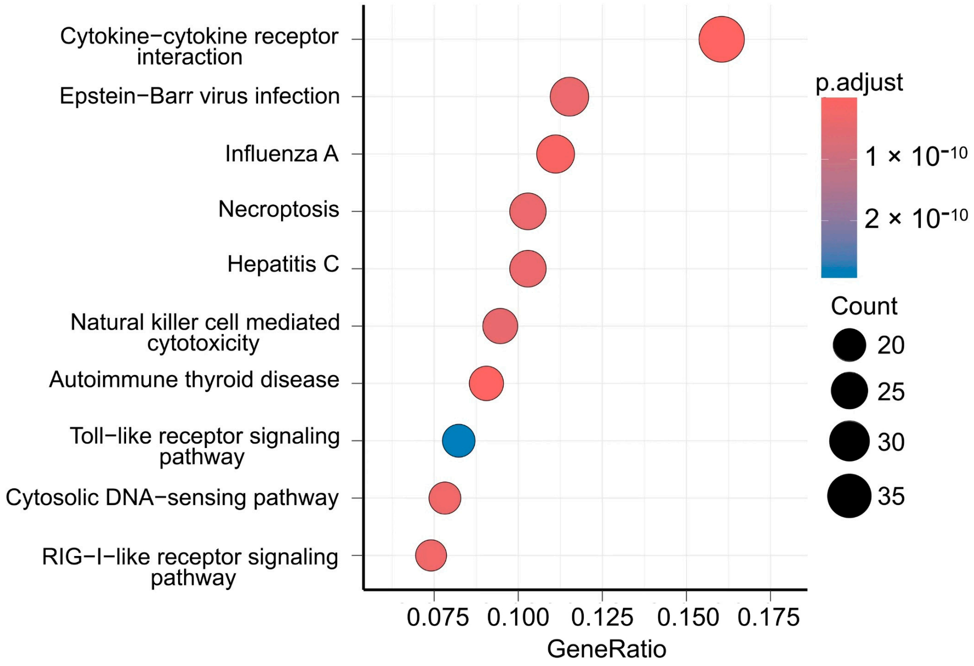

b

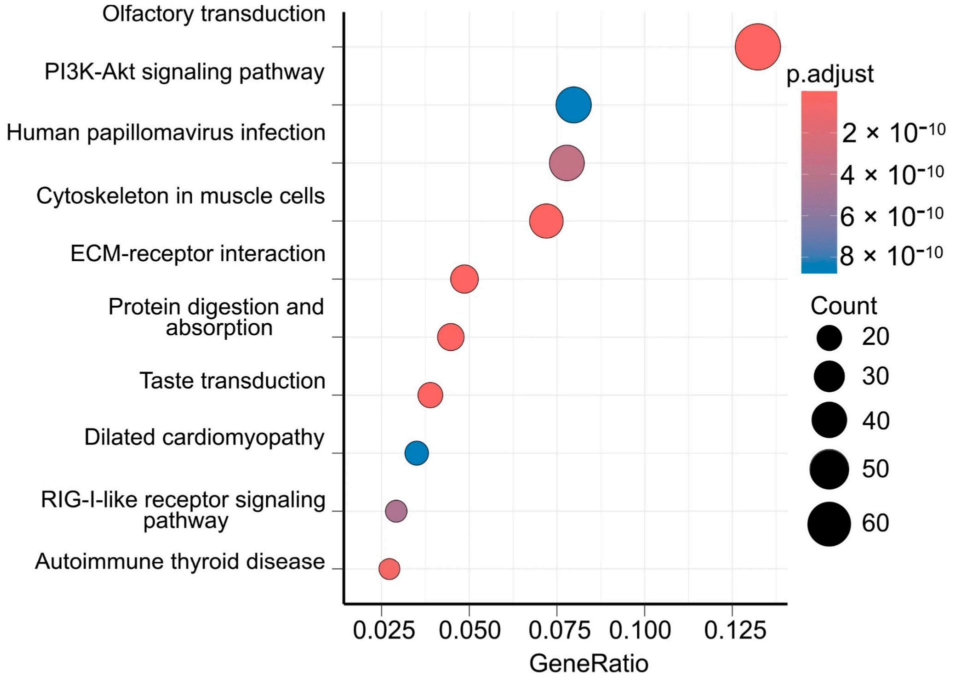

Figure S1. Top 10 KEGG terms of the DEGs (a) Top 10 KEGG terms of the up-DEGs. (b) Top 10 KEGG terms of the down-DEGs.
