# Supplementary material for: Identification of Olfactory Receptors Responding to Androstenone and the Key Structure Determinant in Domestic Pig
Source: Curr Issues Mol Biol. 2024 Dec 30;47(1):13. doi: 10.3390/cimb47010013 (PMC11763519; doi:10.3390/cimb47010013)
Supplement: Supplementary file 1 [file cimb-47-00013-s001.zip › Figure S2.pdf]

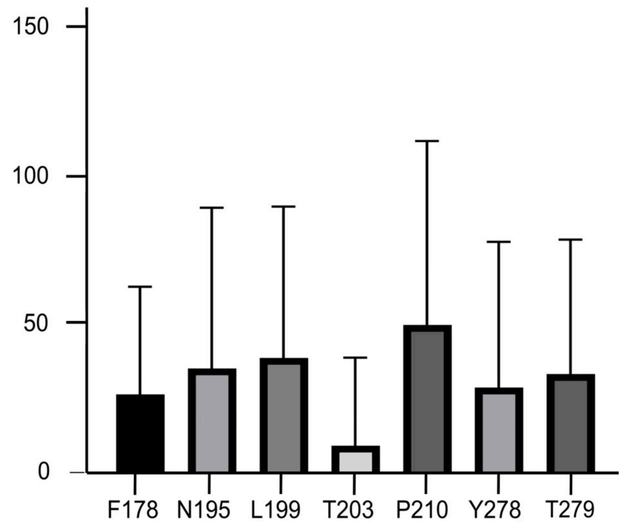

Figure S2. Seven amino acid sites relatively conserved in olfactory receptors responsive to androstenone in binding cavity of pig OR7D4. The vertical coordinate indicates the ratio of the mean Grantham score +1 obtained from a two-by-two comparison of this amino acid site in all annotated ORs to its mean Grantham score +1 obtained from a two-by-two comparison of androstenone -positive ORs.
