# Supplementary material for: Identification of Olfactory Receptors Responding to Androstenone and the Key Structure Determinant in Domestic Pig
Source: Curr Issues Mol Biol. 2024 Dec 30;47(1):13. doi: 10.3390/cimb47010013 (PMC11763519; doi:10.3390/cimb47010013)
Supplement: Supplementary file 1 [file cimb-47-00013-s001.zip › Figure S3.pdf]

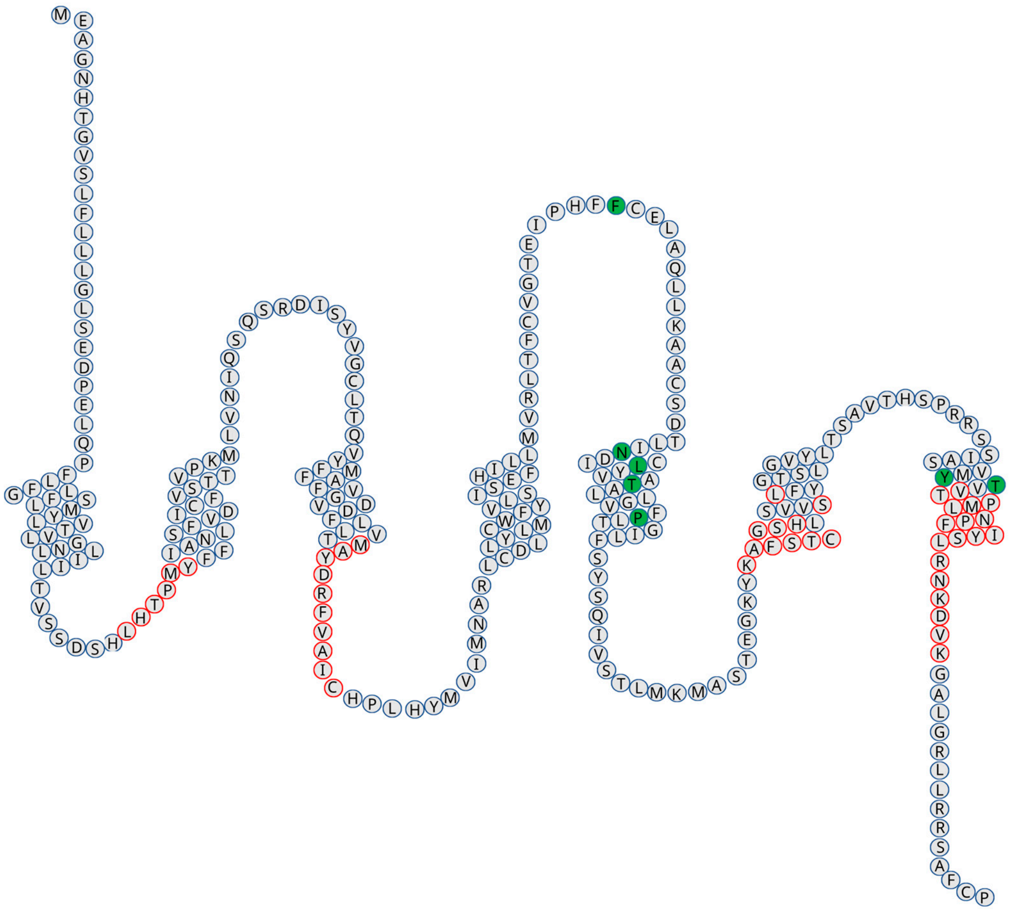

Figure S3. Potential key sites mediating pig OR7D4 response to androstenone. The predicted positions of the 7 key sites on the pig OR7D4 protein. Each small ball represents an amino acid site, with 7 key sites marked in green and conserved motifs in the ORs marked in red boxes.
