# Supplementary material for: Identification of Olfactory Receptors Responding to Androstenone and the Key Structure Determinant in Domestic Pig
Source: Curr Issues Mol Biol. 2024 Dec 30;47(1):13. doi: 10.3390/cimb47010013 (PMC11763519; doi:10.3390/cimb47010013)
Supplement: Supplementary file 1 [file cimb-47-00013-s001.zip › Table S1.pdf]

Table S1. Primers designed for CDS of ORs.

Gene	Primer sequence	Length（bp）
OR2D2	Forward primer: ACGCGTATGAGACGGACAAATCAGACCCA	20
	Reverse primer: GCGGCCGCGCATGGGACATTCCTTGTGGC	20
OR6X1	Forward primer: ACGCGATGAGAAATGGCACTGCAAT	20
	Reverse primer: GCGGCCGCTTACACTGGATGATGAGTCTT	21
OR7D4	Forward primer: ATATACGCGTATGGAAGCAGGAAACCACAC	27
	Reverse primer: ATCTAGGCGGCCGCTCATGGACAAAAGGCTGATC	27
OR8D1	Forward primer: ACGCGATGGTTGCAGGAAATCATTCTAC	20
	Reverse primer: GCGGCCGCTCATTTCCTAACCCAAACCTTC	25
OR8D2	Forward primer: ACGCGATGGCCACTTTAAATCTTTCC	22
	Reverse primer: GCGGCCGCCTACCTTCCCCCAACGACCTT	22
OR10V1	Forward primer: ACGCGATGGAAGAAATAAATAAAACC	21
	Reverse primer: GCGGCCGCTTACATAAGTCCTAAAACCATC	22
OR10Z1	Forward primer: ACGCGATGGGGCAGACCAATGCAA	22
	Reverse primer: GCGGCCGCTCATTTACTCAACAGTCTCCTTCG	22
