# Supplementary material for: Identification of Olfactory Receptors Responding to Androstenone and the Key Structure Determinant in Domestic Pig
Source: Curr Issues Mol Biol. 2024 Dec 30;47(1):13. doi: 10.3390/cimb47010013 (PMC11763519; doi:10.3390/cimb47010013)
Supplement: Supplementary file 1 [file cimb-47-00013-s001.zip › Table S10.pdf]

**Table S10. Ligand-binding cavity residues of pig OR7D4.**

| Amino acids | OR segment position |
|---|---|
| ASN42 | TM1 19 |
| ILE45 | TM1 22 |
| ILE63 | TM2 3 |
| LEU66 | TM2 6 |
| SER67 | TM2 7 |
| ASP70 | TM2 10 |
| PHE73 | TM2 13 |
| THR76 | TM2 16 |
| THR77 | TM2 17 |
| GLN100 | TM3 3 |
| VAL101 | TM3 4 |
| TYR102 | TM3 5 |
| PHE104 | TM3 7 |
| MET105 | TM3 8 |
| ALA106 | TM3 9 |
| PHE107 | TM3 10 |
| VAL108 | TM3 11 |
| GLY109 | TM3 12 |
| ASP111 | TM3 14 |
| ASP112 | TM3 15 |
| PHE113 | TM3 16 |
| LEU115 | TM3 18 |
| VAL151 | TM4 11 |
| VAL152 | TM4 12 |
| ILE155 | TM4 15 |
| SER156 | TM4 16 |
| PHE158 | TM4 18 |
| HIS159 | TM4 19 |
| ILE160 | TM4 20 |
| LEU162 | ECL2 1 |
| MET163 | ECL2 2 |
| LEU166 | ECL2 5 |
| PHE178 | ECL2 17 |

**Table S10. Ligand-binding cavity residues of pig OR7D4 (extend).**

| Amino acids | OR segment position |
|---|---|
| CYS179 | ECL2 18 |
| LEU181 | ECL2 20 |
| ALA182 | ECL2 21 |
| LEU185 | ECL2 24 |
| ASN195 | ECL2 34 |
| CYS198 | TM5 1 |
| LEU199 | TM5 2 |
| ALA202 | TM5 5 |
| THR203 | TM5 6 |
| LEU206 | TM5 9 |
| GLY207 | TM5 10 |
| PRO210 | TM5 13 |
| VAL247 | TM6 7 |
| LEU250 | TM6 10 |
| PHE251 | TM6 11 |
| TYR252 | TM6 12 |
| THR254 | TM6 14 |
| SER255 | TM6 15 |
| TYR278 | TM7.8 |
| THR279 | TM7.9 |
| THR282 | TM7. 12 |
| PRO283 | C' 1 |
| ASN286 | C' 4 |
| PRO287 | C' 7 |
| TYR290 | C' 10 |
