# Supplementary material for: Identification of Olfactory Receptors Responding to Androstenone and the Key Structure Determinant in Domestic Pig
Source: Curr Issues Mol Biol. 2024 Dec 30;47(1):13. doi: 10.3390/cimb47010013 (PMC11763519; doi:10.3390/cimb47010013)
Supplement: Supplementary file 1 [file cimb-47-00013-s001.zip › Table S11.pdf]

Table S11. Missient variant in pig OR7D4.

Variant ID	Nucleobase	Amino acid	Amino acid coordinates
rs703483068	T/C	F/L	26
rs713287097	G/T	G/W	29
rs712114468	C/A	L/M	47
rs697981471	A/G	T/A	48
rs692611647	C/T	P/L	79
rs791922124	C/G	L/V	82
rs705710795	G/A	V/M	83
rs691641734	A/G	Q/R	86
rs701329242	T/G	S/A	87
rs708107540	G/A	R/K	90
rs700399864	G/A	V/I	95
rs696030017	A/G	M/V	105
rs702430735	C/T	A/V	106
rs694959355	G/T	V/F	108
rs704678215	T/C	V/A	108
rs711432320	G/A	V/M	110
rs703836470	C/A	H/N	128
rs696400829	A/G	M/V	133
rs343486793	G/A	R/H	139
rs691515931	C/T	L/F	140
rs698320974	A/C	D/A	142
rs690941402	G/A	V/I	151
rs710156135	G/A	V/I	152
rs692927346	T/C	S/P	154
rs704607334	A/T	M/L	163
rs708367233	G/A	V/M	164
rs694318434	T/C	V/A	164
rs713669102	G/A	R/K	165
rs698208247	C/A	A/D	182
rs706898150	C/A	L/I	184
rs707452579	C/T	T/I	192
rs343067006	G/A	A/T	202
rs326257658	C/T	A/V	202
rs692232368	C/T	T/I	203
rs337710103	C/A	S/Y	219
rs320520502	A/T	T/S	224
rs694240591	C/T	A/V	229
rs331786858	A/G	K/R	236
rs710522888	C/T	T/I	265
rs712648965	C/T	S/F	267
rs335632536	G/T	S/I	271
rs331139591	T/C	V/A	281
