# Supplementary material for: Identification of Olfactory Receptors Responding to Androstenone and the Key Structure Determinant in Domestic Pig
Source: Curr Issues Mol Biol. 2024 Dec 30;47(1):13. doi: 10.3390/cimb47010013 (PMC11763519; doi:10.3390/cimb47010013)
Supplement: Supplementary file 1 [file cimb-47-00013-s001.zip › Table S2.pdf]

Table S2. Mutant primer sequences.

Mutant site	Primer sequence	Primer length
P79L	Forward primer: CACTGTCCtAAAGATGCTAGTGAACATCCAGTCTC	35
	Reverse primer: GCATCTTTaGGACAGTGGTAGAGACGAAGCAG	32
M105G	Forward primer: TgTGGCTTTTGTTGGAGTGGATGATTTCCTCC	32
	Reverse primer: CTCCAACAAAAGCCAcAAAAAAATACACCTGAGTGAGGCA	41
G108F	Forward primer: GGCTTTTtTTGGAGTGGATGATTTCCTCCTGA	32
	Reverse primer: CCACTCCAAaAAAAGCCATAAAAAAATACACCTGAG	37
M133V	Forward primer: TGCACTACgTGGTCATCATGAACGCCCGCCTC	32
	Reverse primer: GATGACCAcGTAGTGCAGGGGGTGGCAGATGG	32
F178A	Forward primer: CACATTTCgcCTGTGAACTGGCTCAGCTTCTCA	33
	Reverse primer: GTTCACAGgcGAAATGTGGGATTTCCGTGCCTA	33
N195A	Forward primer: CTCTCATCgcTGACATCTGTTTGTATGTGGCCA	34
	Reverse primer: AGATGTCAgcGATGAGAGTGTCTGAGCAGGCTG	35
L199A	Forward primer: ACATCTGTgcGTATGTGGCCACTGCCCTGCTGG	34
	Reverse primer: CCACATACgcACAGATGTCATTGATGAGAGTGTCTG	36
A202T	Forward primer: TGTATGTGGtCACTGCCCTGCTGGGTGTGTTT	32
	Reverse primer: GGCAGTGaCCACATACAAACAGATGTCATTGATG	34
A202V	Forward primer: TATGTGGCCAtTGCCCTGCTGGGTGTGTTTCC	33
	Reverse primer: AGGGCAaTGGCCACATACAAACAGATGTCATT	32
T203A	Forward primer: TGTTTGTATGTGGCCgcTGCCCTGCTGGGTGTGTTT	36
	Reverse primer: AgcGGCCACATACAAACAGATGTCATTGATGAG	33
P210A	Forward primer: GTGTGTTTgCTCTCACTGGGATCCTCTTTTCTT	33
	Reverse primer: AGTGAGAGcAAACACACCCAGCAGGGCAGTGG	33
Y278A	Forward primer: TTGCCTCAGTGATGgcCACCGTGGTCACCCCCAT	34
	Reverse primer: TGgcCATCACTGAGGCAATGGAGCTTCTACGGG	33
T279A	Forward primer: TCAGTGATGTACgCCGTGGTCACCCCCATGCT	32
	Reverse primer: ACGGcGTACATCACTGAGGCAATGGAGCTTCT	32
