# Supplementary material for: Identification of Olfactory Receptors Responding to Androstenone and the Key Structure Determinant in Domestic Pig
Source: Curr Issues Mol Biol. 2024 Dec 30;47(1):13. doi: 10.3390/cimb47010013 (PMC11763519; doi:10.3390/cimb47010013)
Supplement: Supplementary file 1 [file cimb-47-00013-s001.zip › Table S3.pdf]

Table S3. Summary of sequencing data.

Sample	Glean Reads	Clean Base (Gb)	Q30 (%)	GC content (%)
Control 1	258882102	38.10	93.36	50.37
Control 2	251804028	36.87	93.51	51.14
Control 3	237809464	35.04	93.34	52.24
Control 4	265404112	38.80	93.85	51.01
Androstenone 1	226943322	33.31	93.44	51.46
Androstenone 2	269669422	39.20	93.97	51.07
Androstenone 3	234642086	34.30	94.50	51.17
Androstenone 4	220055618	32.17	93.61	51.10
