# Supplementary material for: Identification of Olfactory Receptors Responding to Androstenone and the Key Structure Determinant in Domestic Pig
Source: Curr Issues Mol Biol. 2024 Dec 30;47(1):13. doi: 10.3390/cimb47010013 (PMC11763519; doi:10.3390/cimb47010013)
Supplement: Supplementary file 1 [file cimb-47-00013-s001.zip › Table S4.pdf]

Table S4. GO Ontology annotation of upregulated genes in the androstenone treatment group compared to control group.

GO ID	Description	Log (q-value)	Number of genes
GO: 0006959	Humoral immune response	-19.83410991	45
GO: 0033141	Positive regulation of peptidyl-serine phosphorylation of STAT protein	-16.38263234	15
GO: 0002443	Leukocyte mediated immunity	-15.40632335	53
GO: 0033139	Regulation of peptidyl-serine phosphorylation of STAT protein	-15.21546223	15
GO: 0002323	Natural killer cell activation involved in immune response	-14.97619381	17
GO: 0042501	Serine phosphorylation of STAT protein	-14.97619381	15
GO: 0002449	Lymphocyte mediated immunity	-13.422997	44
GO: 0034340	Response to type I interferon	-13.37813615	23
GO: 0060337	Type I interferon-mediated signaling pathway	-13.17126356	22
GO: 0019724	B cell mediated immunity	-13.17126356	33
