# Supplementary material for: Identification of Olfactory Receptors Responding to Androstenone and the Key Structure Determinant in Domestic Pig
Source: Curr Issues Mol Biol. 2024 Dec 30;47(1):13. doi: 10.3390/cimb47010013 (PMC11763519; doi:10.3390/cimb47010013)
Supplement: Supplementary file 1 [file cimb-47-00013-s001.zip › Table S5.pdf]

Table S5. GO Ontology annotation of downregulated genes in the androstenone treatment group compared to control group.

GO ID	Description	Log (q-value)	Number of genes
GO: 0030198	Extracellular matrix organization	-23.68654956	77
GO: 0043062	Extracellular structure organization	-23.68654956	77
GO: 0045229	External encapsulating structure organization	-23.68654956	77
GO: 0001503	Ossification	-20.4743278	85
GO: 0060348	Bone development	-17.85757612	54
GO: 0050907	Detection of chemical stimulus involved in sensory perception	-13.5454501	76
GO: 0060349	Bone morphogenesis	-13.2384799	32
GO: 0030199	Collagen fibril rganization	-12.82424444	26
GO: 0061448	Connective tissue development	-11.47314143	53
GO: 0007608	Sensory perception of smell	-10.61956335	68
