# Supplementary material for: Identification of Olfactory Receptors Responding to Androstenone and the Key Structure Determinant in Domestic Pig
Source: Curr Issues Mol Biol. 2024 Dec 30;47(1):13. doi: 10.3390/cimb47010013 (PMC11763519; doi:10.3390/cimb47010013)
Supplement: Supplementary file 1 [file cimb-47-00013-s001.zip › Table S6.pdf]

Table S6. KEGG pathway enrichment analysis of upregulated genes in the androstenone treatment group compared to control group.

KEGG ID	Description	Log (q-value)	Number of genes
hsa05320	Autoimmune thyroid disease	-18.09	22
has04060	Cytokine-cytokine receptor interaction	-13.77	39
hsa05164	Influenza A	-11.06	27
hsa04622	RIG-I-like receptor signaling pathway	-10.61	18
hsa04623	Cytosolic DNA-sensing pathway	-10.61	19
hsa04217	Necroptosis	-10.51	25
hsa05160	Hepatitis C	-10.51	25
hsa05169	Epstein-Barr virus infection	-10.51	28
hsa04650	Natural killer cell mediated cytotoxicity	-10.44	23
hsa04620	Toll-like receptor signaling pathway	-9.62	20
