# Supplementary material for: Identification of Olfactory Receptors Responding to Androstenone and the Key Structure Determinant in Domestic Pig
Source: Curr Issues Mol Biol. 2024 Dec 30;47(1):13. doi: 10.3390/cimb47010013 (PMC11763519; doi:10.3390/cimb47010013)
Supplement: Supplementary file 1 [file cimb-47-00013-s001.zip › Table S7.pdf]

Table S7. KEGG pathway enrichment analysis of downregulated genes in the androstenone treatment group compared to control group.

KEGG ID	Description	Log (q-value)	Number of genes
hsa04740	Olfactory transduction	-20.82	69
hsa04512	ECM-receptor interaction	-11.71	24
hsa04820	Cytoskeleton in muscle cells	-7.81	37
hsa04974	Protein digestion and absorption	-5.81	23
hsa04742	Taste transduction	-5.44955	20
hsa05320	Autoimmune thyroid disease	-4.26409	14
hsa05165	Human papillomavirus infection	-3.50	40
hsa04622	RIG-I-like receptor signaling pathway	-3.38	15
hsa04151	PI3K-Akt signaling pathway	-3.10	41
