# Supplementary material for: Identification of Olfactory Receptors Responding to Androstenone and the Key Structure Determinant in Domestic Pig
Source: Curr Issues Mol Biol. 2024 Dec 30;47(1):13. doi: 10.3390/cimb47010013 (PMC11763519; doi:10.3390/cimb47010013)
Supplement: Supplementary file 1 [file cimb-47-00013-s001.zip › Table S8.pdf]

Table S8. Downregulated OR genes in the androstenone treatment group compare to control group.

Gene ID	Name	|log2 (FoldChange)|	P value
ENSSSCG00000038356	OR8D2	4.565840098	0.027373691
ENSSSCG00000056646	OR8K5	4.227796248	0.007690141
ENSSSCG00000006447	OR10Z1	3.896149867	0.00520255
ENSSSCG00000011986	OR5K4	3.818111613	0.035178403
ENSSSCG00000031699	OR2T27	3.597245341	0.038938433
ENSSSCG00000030598	OR12D3	3.592534976	0.048621126
ENSSSCG00000037492	OR8D1	3.581020817	0.014082766
ENSSSCG00000025197	OR11H7	3.521132633	0.015417397
ENSSSCG00000061093	OR10A6	3.350041771	0.019640275
ENSSSCG00000031270	OR2D2	3.280979837	0.014184242
ENSSSCG00000055677	OR2M3	3.189755654	0.029710559
ENSSSCG00000013122	OR10V1	3.189714019	0.029713542
ENSSSCG00000014764	OR51E2	3.028408536	0.035834529
ENSSSCG00000015146	OR6X1	2.99469681	0.037501192
ENSSSCG00000055267	OR52B2	2.345334735	0.028907367
