# Supplementary material for: Identification of Olfactory Receptors Responding to Androstenone and the Key Structure Determinant in Domestic Pig
Source: Curr Issues Mol Biol. 2024 Dec 30;47(1):13. doi: 10.3390/cimb47010013 (PMC11763519; doi:10.3390/cimb47010013)
Supplement: Supplementary file 1 [file cimb-47-00013-s001.zip › Table S9.pdf]

Table S9. Downregulated OR-like genes in the androstenone treatment group compared to control group.

Gene ID	|log2(Fold Change)|	P value	Human gene ID	Human gene	Identical to human gene （%）
ENSSSCG00000060054	-5.2268	1.97E-05	ENSG00000284733	*OR4F29*	83.3333
ENSSSCG00000039436	-5.19793	0.001952	ENSG00000198678	*OR5BS1P*	81.3505
ENSSSCG00000046333	-1.94529	0.002453	ENSG00000239590	*OR1J4*	69.0096
ENSSSCG00000058733	-4.04275	0.002566	ENSG00000197454	*OR2L5*	82.3718
ENSSSCG00000058088	-4.82097	0.003528	ENSG00000172362	*OR5B12*	82.1656
ENSSSCG00000058680	-4.52457	0.003567	ENSG00000174667	*OR7D4*	79.4872
ENSSSCG00000056303	-4.24835	0.00419	ENSG00000177275	*OR2AJ1*	75.9146
ENSSSCG00000059167	-3.89615	0.005203	ENSG00000258817	*OR4C13*	81.2298
ENSSSCG00000061943	-3.8045	0.008334	ENSG00000258817	*OR4C13*	79.9353
ENSSSCG00000055049	-3.873	0.010074	ENSG00000181371	*OR5M8*	77.8135
ENSSSCG00000032427	-4.95848	0.0107	ENSG00000204701	*OR2J3*	86.4952
ENSSSCG00000062340	-3.70279	0.012627	ENSG00000239590	*OR1J4*	81.7891
ENSSSCG00000056920	-3.60718	0.012907	ENSG00000205327	*OR6C68*	80.7692
ENSSSCG00000054065	-1.90157	0.013875	ENSG00000260811	*OR4C45*	76.1438
ENSSSCG00000060618	-1.90157	0.013875	ENSG00000260811	*OR4C45*	76.1438
ENSSSCG00000002608	-3.58102	0.014083	ENSG00000130538	*OR11H1*	83.4921
ENSSSCG00000048467	-3.58102	0.014083	ENSG00000184140	*OR4F6*	80.1282
ENSSSCG00000053998	-3.50728	0.014692	ENSG00000260811	*OR4C45*	76.4706
ENSSSCG00000058019	-3.50728	0.014692	ENSG00000260811	*OR4C45*	76.4706
ENSSSCG00000053334	-3.43783	0.016766	ENSG00000197454	*OR2L5*	85.2564
ENSSSCG00000027732	-2.6288	0.016876	ENSG00000239590	*OR1J4*	81.4697
ENSSSCG00000055916	-3.38805	0.0185	ENSG00000188124	*OR2AG2*	78.481
ENSSSCG00000046773	-3.38805	0.0185	ENSG00000197887	*OR1S2*	81.0897
ENSSSCG00000035930	-3.38805	0.0185	ENSG00000174982	*OR4S2*	86.4952
ENSSSCG00000040376	-3.43649	0.018788	ENSG00000155249	*OR4K1*	88.4244
ENSSSCG00000039981	-3.35004	0.01964	ENSG00000258817	*OR4C13*	79.6116
ENSSSCG00000013832	-3.39616	0.025173	ENSG00000127529	*OR7C2*	76.8025
ENSSSCG00000041443	-4.86953	0.025219	ENSG00000169488	*OR4K15*	92.284
ENSSSCG00000031124	-3.2684	0.025635	ENSG00000204701	*OR2J3*	85.209
ENSSSCG00000061665	-4.44704	0.028224	ENSG00000258817	*OR4C13*	78.9644
ENSSSCG00000053476	-4.44704	0.028224	ENSG00000280314	*OR8K3*	70.8333
ENSSSCG00000052015	-3.1365	0.031638	ENSG00000127529	*OR7C2*	77.743
ENSSSCG00000048781	-4.01002	0.032494	ENSG00000182652	*OR4Q3*	82.243
ENSSSCG00000053813	-4.00656	0.032539	ENSG00000182070	*OR52A1*	80.1282
ENSSSCG00000052403	-3.81811	0.035178	ENSG00000244623	*OR2AE1*	81.4241
ENSSSCG00000032913	-3.02841	0.035834	ENSG00000196240	*OR2T2*	86.4198
ENSSSCG00000038894	-3.02841	0.035834	ENSG00000204701	*OR2J3*	88.1029
ENSSSCG00000039603	-3.02841	0.035835	ENSG00000181939	*OR4C15*	68.9873

Table S9. Downregulated OR-like genes in the androstenone treatment group compared to control group (extend).

Gene ID	|log2(Fold Change)|	P value	Human gene stable ID	Human gene	Identical to human gene （%）
ENSSSCG00000033365	-3.02841	0.035835	ENSG00000181939	*OR4C15*	80.0633
ENSSSCG00000031126	-3.00816	0.036799	ENSG00000184140	*OR4F6*	83.9744
ENSSSCG00000033981	-3.43985	0.037009	ENSG00000166884	*OR4D6*	58.9172
ENSSSCG00000062986	-2.9947	0.037501	ENSG00000197454	*OR2L5*	83.9744
ENSSSCG00000037848	-2.9947	0.037501	ENSG00000280236	*OR12D2*	82.7362
ENSSSCG00000052877	-2.9947	0.037501	ENSG00000280236	*OR12D2*	81.1075
ENSSSCG00000035550	-3.59725	0.038938	ENSG00000155249	*OR4K1*	89.3891
ENSSSCG00000048482	-3.59725	0.038938	ENSG00000177201	*OR2T12*	72.1875
ENSSSCG00000060285	-3.5967	0.03896	ENSG00000176495	*OR5AN1*	79.7428
ENSSSCG00000039428	-4.37778	0.03936	ENSG00000212807	*OR2A42*	81.9355
ENSSSCG00000047332	-2.96093	0.040182	ENSG00000197454	*OR2L5*	80.7692
